# Determining magnetic structures in *GSAS-II* using the Bilbao Crystallographic Server tool *k-SUBGROUPSMAG*

**DOI:** 10.1107/S2052520624008436

**Published:** 2024-09-20

**Authors:** Robert B. Von Dreele, Luis Elcoro

**Affiliations:** a Advanced Photon Source Argonne National Laboratory 9700 South Cass Avenue Lemont IL60439-4814 USA; b Department of Physics University of the Basque Country UPV/EHU Apartado 644 48080Bilbao Spain; Brigham Young University, USA

**Keywords:** magnetic subgroup analysis, magnetic structure solution, software

## Abstract

The embedded call to a special version of the web-based Bilbao Crystallographic Server tool *k-SUBGROUPSMAG* from within *GSAS-II* to form a list of all possible commensurate magnetic subgroups of a parent magnetic grey group is described. It facilitates the selection and refinement of the best commensurate magnetic structure model by having all the analysis tools including Rietveld refinement in one place as part of *GSAS-II*. It also provides the chosen magnetic space group as one of the 1421 possible standard Belov–Neronova–Smirnova forms or equivalent non-standard versions.

## Abstract 2

Possible commensurate magnetic structures from neutron powder diffraction data are explored via the Bilbao Crystallographic Server tool *k-SUBGROUPSMAG* called from within *GSAS-II*, which allows easy survey of the results and selection of the best magnetic structure model.

## 1. Introduction

Methods for describing and refining magnetic crystal structures from neutron powder data were embodied in the original Algol and Fortran programs by Rietveld (1969 ▸) and the solution of a magnetic structure at that time was mostly by an *ad hoc* trial-and-error process. A much modified (Wiles & Young, 1981 ▸) descendant of the original Rietveld Fortran code, *FullProf* (Rodríguez-Carvajal, 1993 ▸) makes use of a call to *BasIreps* (Rodríguez-Carvajal, 2010 ▸, 2021 ▸) to develop a list of the possible irreducible representations (irreps) (Bertaut, 1968 ▸) for the magnetic structure. Alternatively, *FullProf* can now use the results of using the web-based tool MAXMAGN (Perez-Mato *et al.*, 2015 ▸), which gives the maximal magnetic space subgroups and can generate a magnetic cif file from a selected entry and the starting paramagnetic structure and a propagation vector. In both cases, the magnetic structure information must be inserted by hand into the *FullProf* control file.

Similarly, the *JANA* system of programs (Dušek *et al.*, 2001 ▸) uses an internal routine to develop irreps and associated magnetic space groups; each is then tested against the data to determine the best description of the magnetic structure.

Here we consider that the symmetry of a commensurate magnetic structure will belong to one of the 1421 possible Belov–Neronova–Smirnova (Belov *et al.*, 1957 ▸) magnetic space groups (Types I, III and IV) just as a commensurate crystal structure uses one of the 230 three-dimensional space groups. The alternative OG description (Opechowski & Guccione, 1965 ▸) is not used here as it does not adhere to a fundamental property of crystal lattices for Type IV magnetic structures, *i.e.* translation symmetry of unit cells. Moreover, the development of magnetic ordering within a crystal structure will have only very minor effects on the parent crystal structure. Thus, the magnetic structure will belong to a subgroup of the corresponding parent structure grey group in which every space-group operation is duplicated by including spin inversion. In addition, extra reflections may be observed that have fractional indices with respect to the parent reciprocal lattice as described by a propagation vector; this expands the available suite of parent magnetic symmetry operations for consideration in forming the proper subgroups for the magnetic structure. Each subgroup is then found by removing a cycle of operations (‘symmetry breaking’) from this suite of operations followed by an appropriate transformation of the remaining operations to have them conform to one of the possible magnetic space groups. This process can be repeated on the subgroups thus found to find lower symmetry subgroups until the lowest magnetic symmetry is reached. Each subgroup must then be tested against the neutron diffraction data to find the best description of the magnetic structure. The web-based Bilbao Crystallographic Server tool, *k-SUBGROUPSMAG* (Perez-Mato *et al.*, 2015 ▸) will do the symmetry breaking analysis beginning with a parent space group and appropriate propagation vector(s) and produce a table of magnetic subgroups or alternatively a graphic display of their hierarchies. Given the parent magnetic ion positions, the Bilbao Crystallographic server tool, *MAGMODELIZE* (Perez-Mato *et al.*, 2015 ▸), will produce the new positions within the selected magnetic subgroup along with the reflection extinction rules. The user must then test these with other software *via* Rietveld refinement (Rietveld, 1969 ▸) against the neutron powder diffraction data obtained from the sample; multiple web calls are required to generate each model each involving selection of relevant web page options. If desired, the corresponding irreps for the subgroup can be found from *k-SUBGROUPSMAG*.

To facilitate these operations, we have embedded a single web call to a special version of *k-SUBGROUPSMAG* in *GSAS-II* (Toby & Von Dreele, 2015 ▸) that returns sufficient data as an html table; this allows the user to select a subgroup and do these all tests directly from within *GSAS-II*. We describe here this implementation and show an example.

## 2. Implementation and example

The general approach for accessing a web page from Python uses the ‘requests.post’ protocol (‘requests’ is a Python package that is part of a normal Python distribution; it must be imported before use). The post command allows defining a Python dictionary of named values that appear within the html code for the web page; then the page returned by the post command is the web site response as if the user had responded to the original web page by filling in data items, pressing buttons, making selections, *etc*. In our hands, the web page html file is parsed from within Python to extract information. The details for this are given in supporting information, Section 1.

As an example, we will show how this works for the antiferromagnetic structure of LaMnO_3_ (paramagnetic LaMnO_3_ space group is *Pnma*) [Moussa *et al.* (1996 ▸) describe it in *Pbnm*]. An inspection of the powder pattern peak indexing shows no evidence of fractional indexed reflections; thus, the propagation vector is (0,0,0).

**Figure 1:**
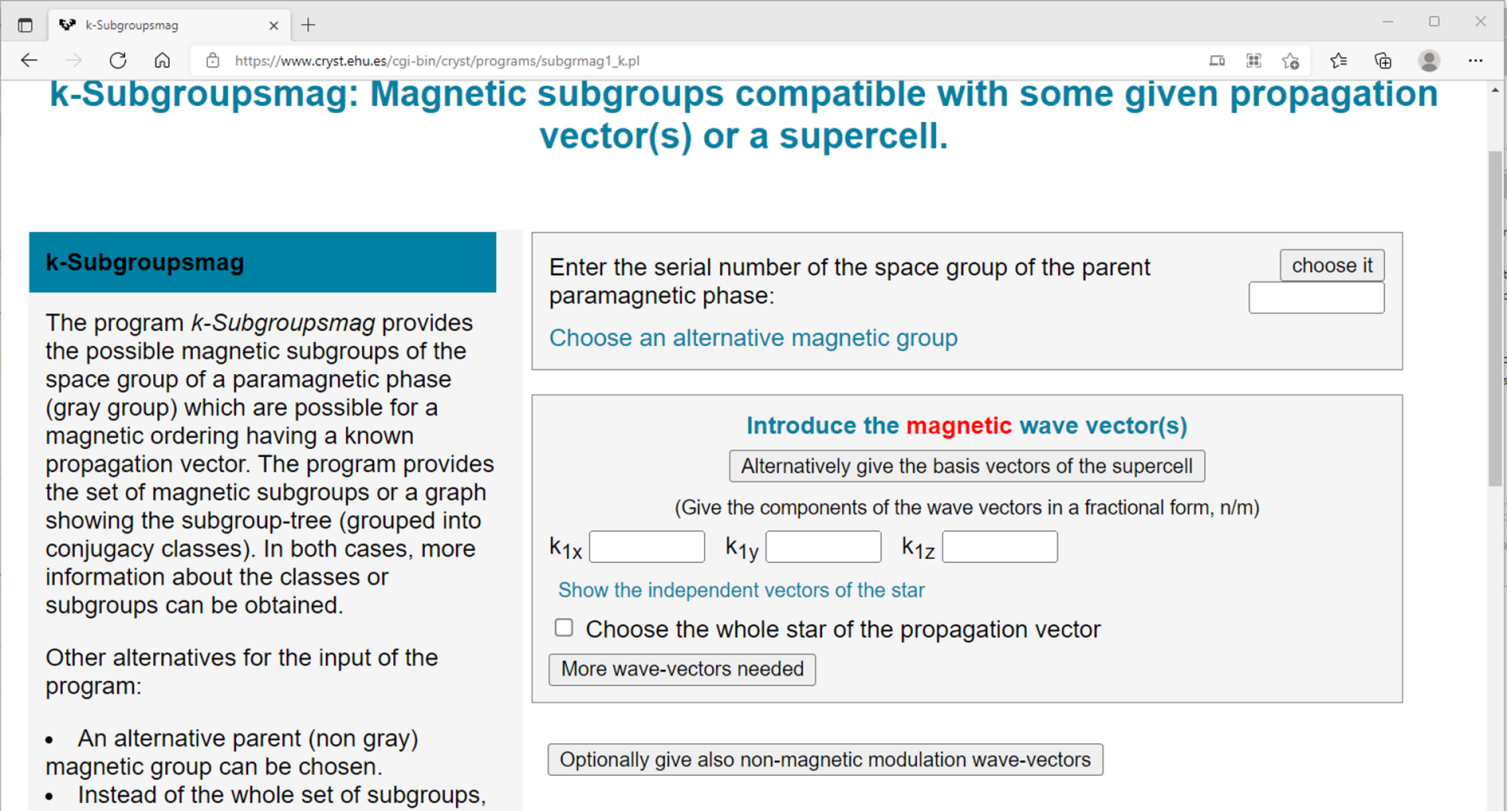
Partial view of the opening screen for *k-SUBGROUPSMAG* as called from the Bilbao Crystallographic Server/Magnetic Symmetry and Applications web site.

**Figure 2:**
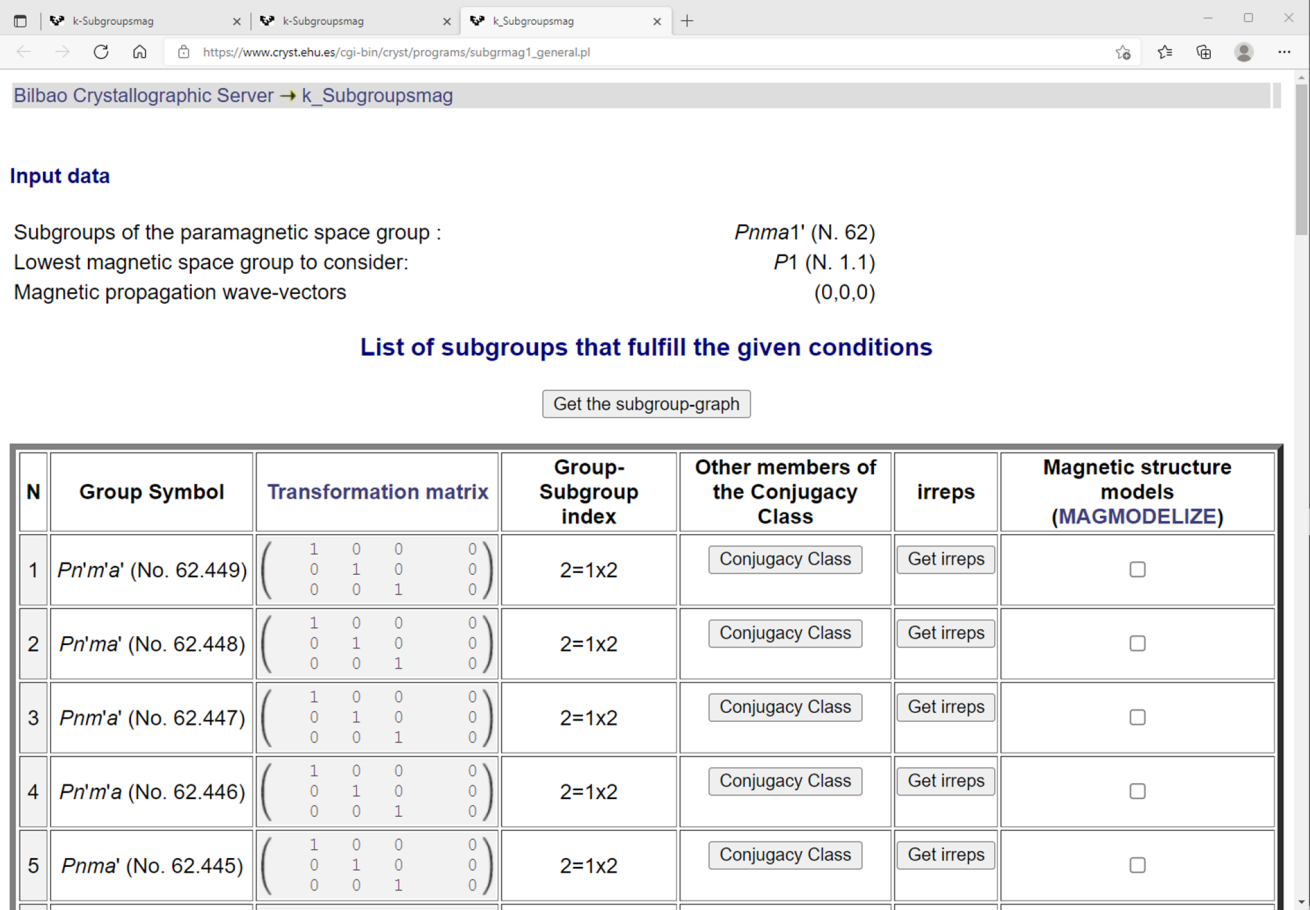
Partial view of the response from *k-SUBGROUPSMAG* for the grey group *Pnma*1′ with propagation vector = (0,0,0).

For *k-SUBGROUPSMAG* (https://www.cryst.ehu.es/cgi-bin/cryst/programs/subgrmag1_k.pl), the returned web page looks, in part, like that shown in Fig. 1 ▸. Normally, the user would select a space group for the parent paramagnetic structure, enter propagation vector(s) and make some optional choices and finally press the ‘submit’ button at the bottom of the screen (not shown). For LaMnO_3_, we would select *Pnma* (serial No. 62) as the parent paramagnetic phase and set the propagation vector to (0,0,0); *k-SUBGROUPSMAG* would return a web page showing a table of 51 magnetic subgroups (Fig. 2 ▸ shown in part for parent *Pnma*1′ grey space group).

The user would then select one (or more) for further study *viaMAGMODELIZE* which uses a cif file of the parent structure provided by the user to create a magnetic structure model as a new cif file to try via a Rietveld refinement. This process is effective in solving the magnetic structure but involves many interactions with the magnetic structure utilities in the Bilbao site combined with Rietveld refinement tests of each model to be tested.

**Figure 3:**
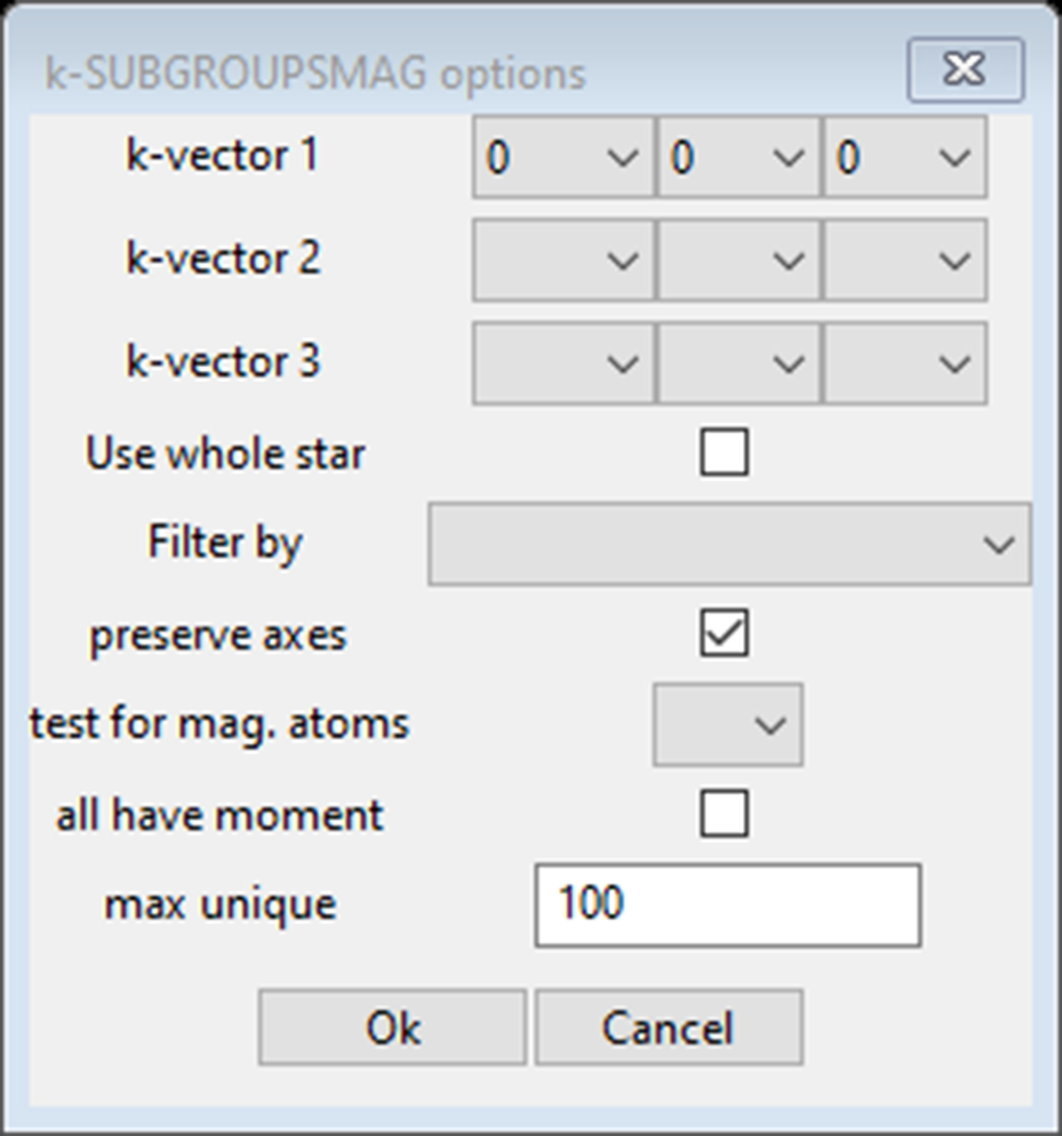
Pop-up window from *GSAS-II* requesting data from the user for the special *GSAS-II* version of *k-SUBGROUPSMAG*.

For the *GSAS-II* implementation of this, the user begins with a *GSAS-II* project that has defined the parent paramagnetic phase (most likely from a previous Rietveld refinement with data taken above the magnetic ordering temperature) which has the full structural details (space group, unit-cell parameters and atom positions) already defined. Then *GSAS-II* shows a simple request (Fig. 3 ▸) that mirrors what was asked directly by *k-SUBGROUPSMAG* and includes the selection of a magnetic atom (in our case Mn).

**Figure 4:**
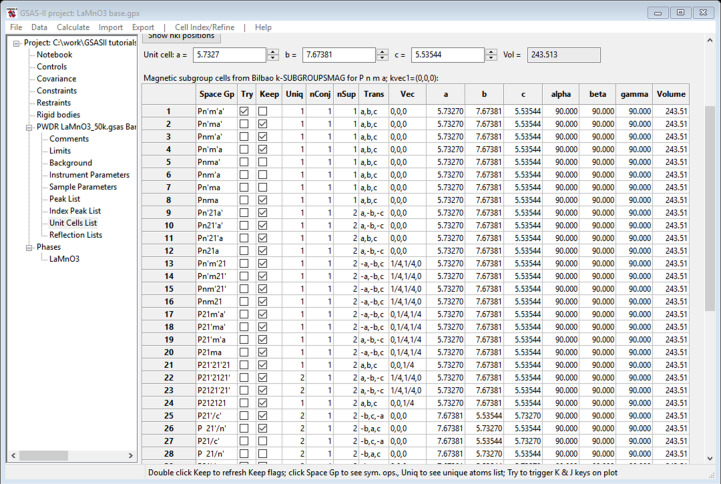
Table of magnetic subgroups of *Pnma*1′ shown by *GSAS-II* as obtained from the special version of *k-SUBGROUPSMAG*.

With this information, a special version of *k-SUBGROUPSMAG* (https://www.cryst.ehu.es/cgi-bin/cryst/programs/subgrmag1_general_GSAS.pl?) is called. It returns an html table that is parsed by *GSAS-II* (see supporting information, Section 1 for details) to produce a list of all the subgroups that are possible (Fig. 4 ▸)

**Figure 5:**
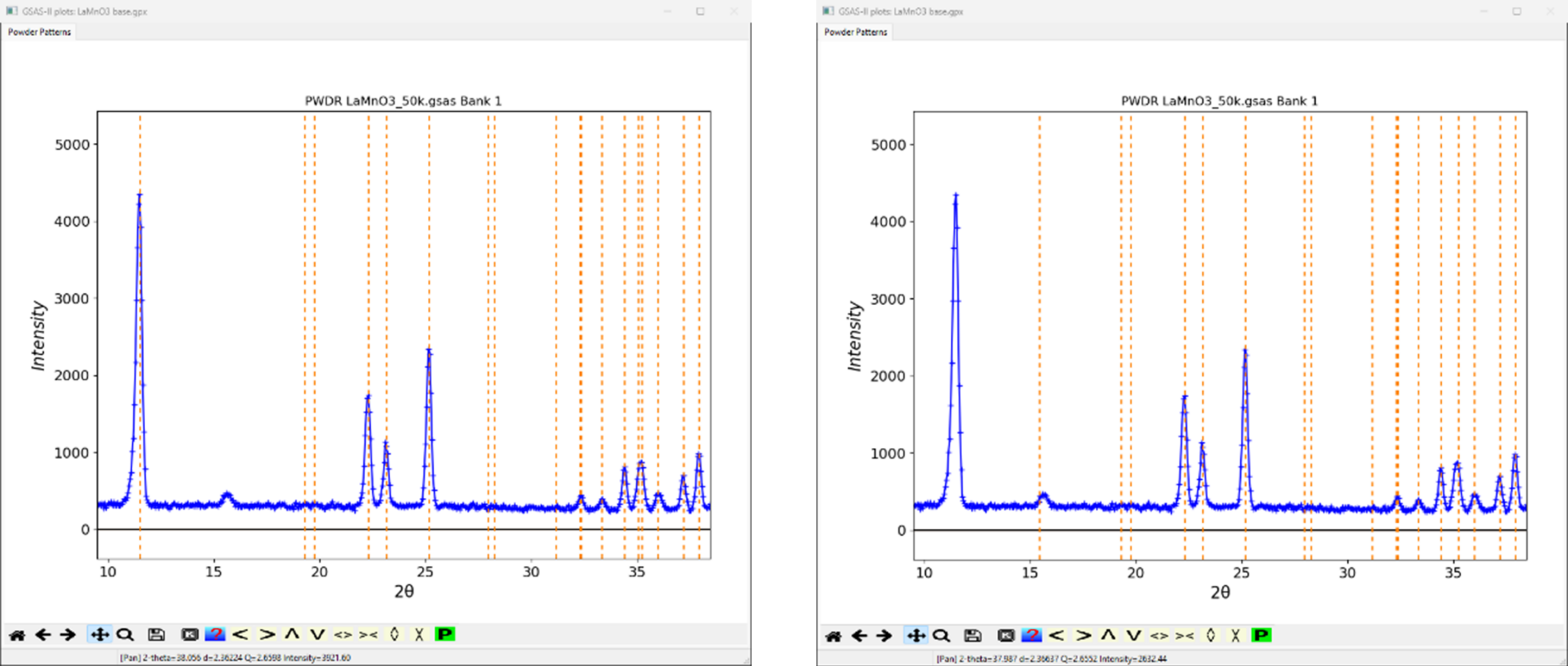
Indexing comparison: *Pn*′*ma*′ (No. 2 in Fig. 4 ▸) on left versus *Pnm*′*a*′ (No. 3 in Fig. 4 ▸) on right. In both the vertical dashed lines are expected Bragg peaks for the indicated magnetic space group. The small peak at ∼16° 2θ is from a contaminating phase. LaMnO_3_ powder data from NIST BT1 diffractometer kindly obtained from Q. Huang.

The overall hierarchy of this table is that those subgroups that result from loss of a single cycle of operations (‘maximal’ subgroups) are shown first; in many cases the correct subgroup is found here as they conform to a single Landau-type magnetic transition. The next sets are for loss of additional cycles of operations. In this case, they will be in the sequence orthorhombic → monoclinic → triclinic subgroups. One rarely must resort to one of these for the correct subgroup. To facilitate selection of viable solutions to the magnetic structure, this table shows for each subgroup the number of unique magnetic atoms and if a non-zero moment is permitted by symmetry (the ‘Keep’ column). This was determined by *GSAS-II* from the special position magnetic moment symmetry constraints for the Mn atoms transformed to their subgroup positions (via Trans and Vec in Fig. 4 ▸). Some of the subgroups use nonstandard (*e.g.* rows 9–12 in Fig. 4 ▸) space group symbols so the unit-cell parameters can better match the parent unit cell (in contrast, *k-SUBGROUPSMAG* shows them only in the standard version of the subgroup symbol). The ‘Try’ column allows one to visually check the reflection indexing for any magnetic subgroup against the powder pattern. For this example, there are two possibilities (Nos. 2 and 8 in Fig. 4 ▸) within the first eight magnetic subgroups; they both index every reflection seen in the powder pattern. Note that Nos. 1, 5, 6 and 7 in Fig. 4 ▸ do not allow magnetic moments on the Mn atom position (‘Keep’ flag unchecked); and Nos. 3 and 4 do not correctly index the powder pattern. Fig. 5 ▸ compares indexing with *Pn*′*ma*′ (No. 2 in Fig. 4 ▸) and *Pnm*′*a*′ (No. 3 in Fig. 4 ▸); the latter does not index the large peak at ∼12° 2θ.

**Figure 6:**
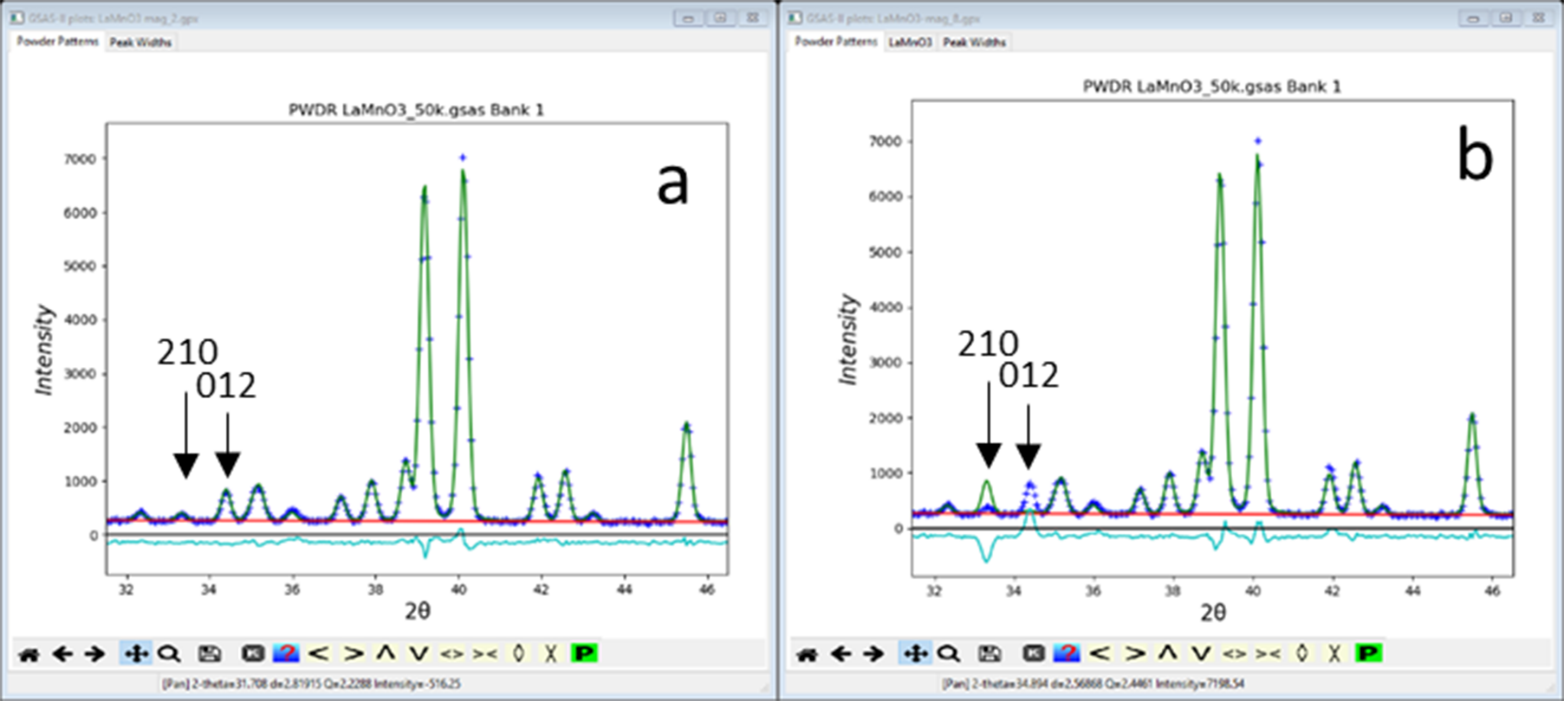
Screen shots from *GSAS-II* showing a selected part of the Rietveld refinement fit for the LaMnO_3_ (*a*) *Pn*′*ma*′ and (*b*) *Pnma* magnetic structures. Two reflections (210 and 012) are marked. In each plot the ’+’ mark the observed data, green line is that calculated from their respective best fit model, the red line is the calculated background from the fit and the cyan line below is the (*I*_obs_ − *I*_calc_) difference.

In *GSAS-II*, the process for solving the structure continues by selecting ‘Keep’ entries in turn for Rietveld refinement; new *GSAS-II* project files are created for each. *GSAS-II* produces a model with two phases, one for the full chemical structure and the other for just the magnetic ions. They are automatically connected by appropriate constraints between all common parameters to keep them in sync during refinement. In this example, No. 2 (*Pn*′*ma*′) gave a better result (lower *R*_wp_ = 6.28%) than No. 8 (*Pnma*; *R*_wp_ = 8.39%). In each case only one moment component was found to be non-zero; the others were thus constrained to be zero. The most evident differences between the two fits are easily seen in their respective powder patterns [Fig. 6 ▸(*a*) for No. 2 *Pn*′*ma*′ and 6 ▸(*b*) for No. 8 *Pnma*] as displayed in *GSAS-II*.

**Figure 7:**
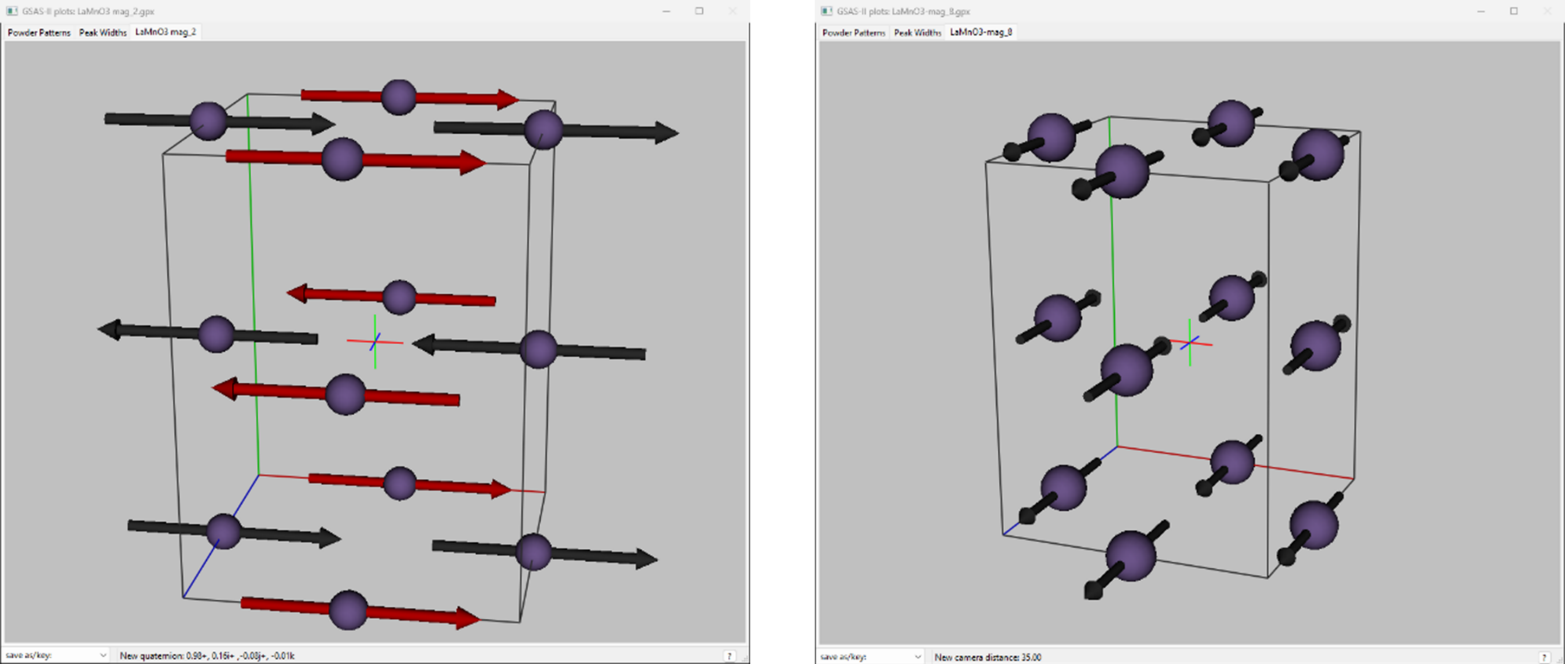
Screen shots from *GSAS-II* showing the LaMnO_3_ magnetic structures obtained for (left) subgroup *Pn*′*ma*′ (No. 2) and (right) subgroup *Pnma* (No. 8). Red arrows mark those moments generated via spin inversion operations. The axes edges are coloured red, blue and green for the *a*, *b* and *c* axes, respectively.

In particular, the reflection pair, 210 and 012, are well fitted with the *Pn*′*ma*′ model [Fig. 6 ▸(*a*)] but not by the *Pnma* one [Fig. 6 ▸(*b*)], where the calculated 210 reflection is too strong and the calculated 012 reflection is too weak relative to their respective observed intensities. Finally, one can draw the two magnetic structures directly from within *GSAS-II* to see what the difference is (Fig. 7 ▸).

Both structures are antiferromagnetic, but the correct one (No. 2 *Pn*′*ma*′) has the moment directions parallel to the crystallographic *a* axis while the incorrect one (No. 8 *Pnma*) has them parallel to the *b* axis. This result compares well with the determination by Moussa *et al.* (1996 ▸) except that here the structure is described with respect to the *Pnma* parent space group and here the magnetic space group is identified as *Pn*′*ma*′; Moussa *et al.* (1996 ▸) did not identify the magnetic space group.

## 3. Conclusion

The inclusion of a single call to a special version of the Bilbao Crystallographic Server tool, *k-SUBGROUPSMAG*, into *GSAS-II* that generates all the magnetic subgroups of a parent grey group and propagation vector provides a straightforward method for solving and refining commensurate magnetic structures. The result explicitly identifies the Belov–Neronova–Smirnova magnetic space group for the structure, which can be either one of the 1421 standard Belov–Neronova–Smirnova groups or as a possibly more suitable non-standard equivalent one.

## Supplementary Material

Supporting information file. DOI: 10.1107/S2052520624008436/cam5001sup1.pdf
