# Supplementary material for: Determining magnetic structures in GSAS-II using the Bilbao Crystallographic Server tool k-SUBGROUPSMAG
Source: Acta Crystallogr B Struct Sci Cryst Eng Mater. 2024 Sep 20;80(Pt 5):424–9. doi: 10.1107/S2052520624008436 (PMC11457102; doi:10.1107/S2052520624008436)
Supplement: Supplementary file 1 [file b-80-00424-sup1.pdf]

Acta Cryst B

STRUCTURAL SCIENCE
CRYSTAL ENGINEERING
MATERIALS

Volume 80 (2024)

Supporting information for article:

Determining magnetic structures in GSAS-II using the Bilbao Crystallographic Server tool *k-SUBGROUPSMAG*

Robert B. Von Dreele and Luis Elcoro

S1. The use of python requests.post in GSAS-II for access of special version of k-SUBGROUPSMAG

Once the requests python package has been imported into a python session, requests.post can be used as

Output = requests.post(URL, parmdict)

In this case the URL is https://www.cryst.ehu.es/cgi-bin/cryst/programs/subgrmag1_general_GSAS.pl? and parmdict is the python dictionary shown in Figure S1. The value items were found (and confirmed by L. Elcoro) by examination of the html code returned by a call to k-SUBGROUPSMAG (Figure 1). The only values set within GSAS-II are: 'landau' (if 'yes' then the subgroups are limited to those with a single irrep parameter, 'limite' (if 'maximal' then only maximal subgroups are returned, otherwise 'spgroup' for all subgroups returned), 'starmag' (if 'yes' then use the star of propagation vectors), 'km1x', 'km1y','km1z' (propagation vector components as strings; there can be 3 sets), and 'generators' (a single string with all the parent space group operators separated by new line character '\n'). This use of generators allows the use of a non-standard setting of the parent space group by this version of k-SUBGROUPSMAG.

```
parmdict = {'centrosymmetry': '0', 'crystalsystem': '0', 'landau': 'no', 'eleccion': 'subgrmag1_k',
 'inicio': 'nostandard', 'celtodas': 'no', 'limite': 'spgroup', 'list': 'Submit', 'listado': 'lista',
'starmagnetica': 'no', 'pointgroup': '0', 'polarity': '0', 'sub': '1.1', 'super': '', 'tipog': 'gmag',
'wyckoffstrain': '', 'km1x': '0', 'km1y': '0', 'km1z': '0',
'generators': 'x,y,z\n-x+1/2,y+1/2,z+1/2\nx,-y+1/2,z\n-x+1/2,-y,z+1/2\n-x,-y,-z\nx+1/2,-y+1/2,-
z+1/2\n-x,y+1/2,-z\nx+1/2,y,-z+1/2'}
```

Figure S1 Python dictionary of parameters needed for requests.post call to https://www.cryst.ehu.es/cgi-bin/cryst/programs/ subgrmag1_general_GSAS.pl?

Part of the return html page, Output, from requests.post is shown in Figure S2; subsequent python steps convert this single (large) string into a list of substrings, one for each subgroup. This is shown in Figure S3. Each line consists of 2 indices (the second is in rank order), an integer (used only if star is requested), the BNS magnetic space group number, the BNS magnetic space group symbol, the 3 rows of the parent to subgroup transformation matrix, the origin shift vector, a list of supergroups in the list for this subgroup and the group-subgroup index.

"62.442tyesz{{1,0,0},{0,1,0},{0,0,1}}z{0,0,0}&7z1.1z0z62.449z<i>P</i><i>n</i>'<i>m</i>'<i
>a</i>'z{{1,0,0},{0,1,0},{0,0,1}}z{0,0,0}z{0}z2=1x2&4z2.1z0z62.448z<i>P</i><i>n</i>'<i>
m</i><i>a</i>'z{{1,0,0},{0,1,0},{0,0,1}}z{0,0,0}z{0}z2=1x2&6z3.1z0z62.447z<i>P</i><i>n
</i><i>m</i>'<i>a</i>'z{{1,0,0},{0,1,0},{0,0,1}}z{0,0,0}z{0}z2=1x2&2z4.1z0z62.446z<i>P<
/i><i>n</i>'<i>m</i>'<i>a</i>z{{1,0,0},{0,1,0},{0,0,1}}z{0,0,0}z{0}z2=1x2&1z5.1z0z62.445
z<i>P</i><i>n</i><i>m</i><i>a</i>'z{{1,0,0},{0,1,0},{0,0,1}}z{0,0,0}z{0}z2=1x2&3z6.1z0z
62.444z<i>P</i><i>n</i><i>m</i>'<i>a</i>z{{1,0,0},{0,1,0},{0,0,1}}z{0,0,0}z{0}z2=1x2&5z
7.1z0z62.443z<i>P</i><i>n</i>'<i>m</i><i>a</i>z{{1,0,0},{0,1,0},{0,0,1}}z{0,0,0}z{0}z2=1
x2&8z8.1z0z62.441z<i>P</i><i>n</i><i>m</i><i>a</i>z{{1,0,0},{0,1,0},{0,0,1}}z{0,0,0}z{0
}z2=1x2…
…47z50.1z0z2.4z<i>P</i>-
1z{{1,0,0},{0,1,0},{0,0,1}}z{0,0,0}z{18,19,20,25,30,35}z8=1x8&51z51.1z0z1.1z<i>P</i>1z{
{1,0,0},{0,1,0},{0,0,1}}z{0,0,0}z{37,38,39,40,41,42,43,44,45,46,47,48,49,50}z16=1x16"

Figure S2 Part of the output html page from the request.post call to https://www.cryst.ehu.es/cgi-bin/cryst/programs/ subgrmag1_general_GSAS.pl?. This is a single string and the symbol '&' indicates a new line and the 'z' symbol separates each line into data items.

7,1.1,0,62.449,Pn'm'a',{{1,0,0},{0,1,0},{0,0,1}},{0,0,0},{0},2=1x2
4,2.1,0,62.448,Pn'ma',{{1,0,0},{0,1,0},{0,0,1}},{0,0,0},{0},2=1x2
6,3.1,0,62.447,Pnm'a',{{1,0,0},{0,1,0},{0,0,1}},{0,0,0},{0},2=1x2
2,4.1,0,62.446,Pn'm'a,{{1,0,0},{0,1,0},{0,0,1}},{0,0,0},{0},2=1x2
1,5.1,0,62.445,Pnma',{{1,0,0},{0,1,0},{0,0,1}},{0,0,0},{0},2=1x2
3,6.1,0,62.444,Pnm'a,{{1,0,0},{0,1,0},{0,0,1}},{0,0,0},{0},2=1x2
5,7.1,0,62.443,Pn'ma,{{1,0,0},{0,1,0},{0,0,1}},{0,0,0},{0},2=1x2
8,8.1,0,62.441,Pnma,{{1,0,0},{0,1,0},{0,0,1}},{0,0,0},{0},2=1x2
26,9.1,0,33.148,Pn'a'2_1,{{1,0,0},{0,0,-1},{0,1,0}},{0,0,0},{4,7},4=1x4
16,10.1,0,33.147,Pna'2_1',{{1,0,0},{0,0,-1},{0,1,0}},{0,0,0},{1,6},4=1x4
10,11.1,0,33.146,Pn'a2_1',{{1,0,0},{0,0,1},{0,-1,0}},{0,0,0},{2,5},4=1x4
29,12.1,0,33.144,Pna2_1,{{1,0,0},{0,0,-1},{0,1,0}},{0,0,0},{3,8},4=1x4
21,13.1,0,31.127,Pm'n'2_1,{{0,-1,0},{1,0,0},{0,0,1}},{1/4,1/4,0},{2,7},4=1x4
12,14.1,0,31.126,Pmn'2_1',{{0,-1,0},{1,0,0},{0,0,1}},{1/4,1/4,0},{4,5},4=1x4
15,15.1,0,31.125,Pm'n2_1',{{0,-1,0},{1,0,0},{0,0,1}},{1/4,1/4,0},{3,6},4=1x4
24,16.1,0,31.123,Pmn2_1,{{0,-1,0},{1,0,0},{0,0,1}},{1/4,1/4,0},{1,8},4=1x4
31,17.1,0,26.70,Pm'c'2_1,{{0,-1,0},{0,0,-1},{1,0,0}},{0,1/4,1/4},{6,7},4=1x4
14,18.1,0,26.69,Pmc'2_1',{{0,-1,0},{0,0,-1},{1,0,0}},{0,1/4,1/4},{1,4},4=1x4
11,19.1,0,26.68,Pm'c2_1',{{0,-1,0},{0,0,1},{-1,0,0}},{0,1/4,1/4},{2,3},4=1x4
34,20.1,0,26.66,Pmc2_1,{{0,-1,0},{0,0,-1},{1,0,0}},{0,1/4,1/4},{5,8},4=1x4
23,21.1,0,19.27,P2_1'2_1'2_1,{{1,0,0},{0,1,0},{0,0,1}},{0,0,1/4},{1,2},4=1x4
28,22.1,0,19.27,P2_1'2_1'2_1,{{1,0,0},{0,0,1},{0,-1,0}},{1/4,1/4,0},{3,4},4=1x4

33,23.1,0,19.27,$P2_1'2_1'2_1$,{{0,0,-1},{0,1,0},{1,0,0}},{1/4,1/4,0},{5,6},4=1x4
36,24.1,0,19.25,$P2_12_12_1$,{{1,0,0},{0,1,0},{0,0,1}},{0,0,1/4},{7,8},4=1x4
18,25.1,0,14.79,$P2_1'/c'$,{{0,-1,0},{0,0,-1},{1,0,0}},{0,0,0},{4,6},4=1x4
20,26.1,0,14.79,$P2_1'/c'$,{{0,0,-1},{1,0,0},{0,-1,1}},{0,0,0},{2,4},4=1x4
22,27.1,0,14.78,$P2_1/c'$,{{0,-1,0},{0,0,-1},{1,0,0}},{0,0,0},{1,7},4=1x4
32,28.1,0,14.78,$P2_1/c'$,{{0,0,-1},{1,0,0},{0,-1,1}},{0,0,0},{5,7},4=1x4
9,29.1,0,14.77,$P2_1'/c$,{{0,-1,0},{0,0,-1},{1,0,0}},{0,0,0},{3,5},4=1x4
17,30.1,0,14.77,$P2_1'/c$,{{0,0,-1},{1,0,0},{0,-1,1}},{0,0,0},{1,3},4=1x4
25,31.1,0,14.75,$P2_1/c$,{{0,-1,0},{0,0,-1},{1,0,0}},{0,0,0},{2,8},4=1x4
35,32.1,0,14.75,$P2_1/c$,{{0,0,-1},{1,0,0},{0,-1,1}},{0,0,0},{6,8},4=1x4
19,33.1,0,11.54,$P2_1'/m'$,{{1,0,0},{0,1,0},{0,0,1}},{0,0,0},{2,6},4=1x4
27,34.1,0,11.53,$P2_1/m'$,{{1,0,0},{0,1,0},{0,0,1}},{0,0,0},{3,7},4=1x4
13,35.1,0,11.52,$P2_1'/m$,{{1,0,0},{0,1,0},{0,0,1}},{0,0,0},{1,5},4=1x4
30,36.1,0,11.50,$P2_1/m$,{{1,0,0},{0,1,0},{0,0,1}},{0,0,0},{4,8},4=1x4
37,37.1,0,7.26,Pc',{{0,-1,0},{0,0,-1},{1,0,0}},{0,0,1/4},{14,16,18,22,26,31},8=1x8
39,38.1,0,7.26,Pc',{{0,0,-1},{1,0,0},{0,-1,1}},{1/4,0,0},{10,12,20,21,26,32},8=1x8
44,39.1,0,7.24,Pc,{{0,-1,0},{0,0,-1},{1,0,0}},{0,0,1/4},{9,10,11,25,29,34},8=1x8
46,40.1,0,7.24,Pc,{{0,0,-1},{1,0,0},{0,-1,1}},{1/4,0,0},{15,16,17,24,29,35},8=1x8
38,41.1,0,6.20,Pm',{{1,0,0},{0,1,0},{0,0,1}},{0,1/4,0},{11,15,19,21,27,31},8=1x8
45,42.1,0,6.18,Pm,{{1,0,0},{0,1,0},{0,0,1}},{0,1/4,0},{12,13,14,24,30,34},8=1x8
41,43.1,0,4.9,$P2_1'$,{{1,0,0},{0,0,1},{0,-1,0}},{1/4,0,0},{9,12,15,18,28,33},8=1x8
42,44.1,0,4.9,$P2_1'$,{{1,0,0},{0,1,0},{0,0,1}},{0,0,0},{10,13,16,19,23,33},8=1x8
43,45.1,0,4.9,$P2_1'$,{{0,1,0},{-1,0,0},{0,0,1}},{0,1/4,1/4},{11,14,17,20,23,28},8=1x8
48,46.1,0,4.7,$P2_1$,{{1,0,0},{0,0,1},{0,-1,0}},{1/4,0,0},{21,22,23,24,25,36},8=1x8
49,47.1,0,4.7,$P2_1$,{{1,0,0},{0,1,0},{0,0,1}},{0,0,0},{26,27,28,29,30,36},8=1x8
50,48.1,0,4.7,$P2_1$,{{0,1,0},{-1,0,0},{0,0,1}},{0,1/4,1/4},{31,32,33,34,35,36},8=1x8
40,49.1,0,2.6,P-1',{{1,0,0},{0,1,0},{0,0,1}},{0,0,0},{9,13,17,22,27,32},8=1x8
47,50.1,0,2.4,P-1,{{1,0,0},{0,1,0},{0,0,1}},{0,0,0},{18,19,20,25,30,35},8=1x8
51,51.1,0,1.1,P1,{{1,0,0},{0,1,0},{0,0,1}},{0,0,0},{37,38,39,40,41,42,43,44,45,46,47,48,49,50},16=1x16

Figure S3 The full list of 51 subgroup entries taken from the html file output from request.post call to https://www.cryst.ehu.es/cgi-bin/cryst/programs/ subgrmag1_general_GSAS.pl?.

Each entry is processed by GSAS-II to make the corresponding entry in Figure 4; the parent lattice parameters and magnetic ion coordinates are transformed to the subgroup lattice. The magnetic ion positions are checked for magnetic site symmetry and if any moment components are allowed, the ‘Keep’ box for that subgroup is checked.
